# Brain glucose sensing, glucokinase and neural control of metabolism and islet function

**DOI:** 10.1111/dom.12334

**Published:** 2014-09-09

**Authors:** E O Ogunnowo-Bada, N Heeley, L Brochard, M L Evans

**Affiliations:** Wellcome Trust-MRC Institute of Metabolic Science, IMS Metabolic Research Laboratories, University of CambridgeCambridge, UK

**Keywords:** glucokinase, glucose sensing, glucose-stimulated insulin secretion, hypoglycaemia, hypothalamus, insulin

## Abstract

It is increasingly apparent that the brain plays a central role in metabolic homeostasis, including the maintenance of blood glucose. This is achieved by various efferent pathways from the brain to periphery, which help control hepatic glucose flux and perhaps insulin-stimulated insulin secretion. Also, critically important for the brain given its dependence on a constant supply of glucose as a fuel – emergency counter-regulatory responses are triggered by the brain if blood glucose starts to fall. To exert these control functions, the brain needs to detect rapidly and accurately changes in blood glucose. In this review, we summarize some of the mechanisms postulated to play a role in this and examine the potential role of the low-affinity hexokinase, glucokinase, in the brain as a key part of some of this sensing. We also discuss how these processes may become altered in diabetes and related metabolic diseases.

## Introduction

In health, blood glucose is maintained within relatively narrow limits despite a variety of potential perturbations. This control is effected by co-ordinated homeostatic physiological responses in pancreas, liver and skeletal muscle. Over recent years, it has become apparent that the brain also plays a pivotal role in controlling peripheral energy and metabolic homeostasis, including the control of blood glucose. Teleologically, this would appear to be logical given the dependence of the brain on a constant uninterrupted supply of glucose from the blood stream to fuel metabolism and support function.

A key part of this homeostatic process is that the brain needs to detect rapidly and accurately changes in blood glucose. Specialized brain fuel sensors monitor changes in ambient glucose. Importantly, in addition to local fuel sensing, the brain also receives information about changes in blood glucose from a number of other sources. Levels of circulating hormones such as insulin and glucagon respond to changes in blood glucose. Afferent neural inputs from peripheral sites – for example, vagal afferents from hepatic portal glucose sensors also feed in information.

The brain then utilizes this information to exert central and strategic control on a number of aspects of metabolism and energy balance. Triggering of counter-regulatory responses to falling glucose is perhaps the most obvious exemplar, but autonomic outflow from the brain also helps control hepatic glucose flux and facilitates glucose-stimulated insulin release from the pancreas.

A number of candidate mechanisms and indeed brain areas and cell types have been postulated as being important for local brain glucose sensing. We review below the current data, focusing particularly on the evidence for a role for the low-affinity hexokinase, glucokinase, in brain. In this review, we will: (i) first provide an outline of the evidence that brain glucose sensing is important for glucose homeostasis, (ii) give an overview of brain glucose-sensing mechanisms including those using glucokinase, (iii) then describe the current data supporting a role for brain glucokinase in the central control of glucose homeostasis and (iv) discuss how these processes may become altered in diabetes and related metabolic disorders.

## Brain Glucose Sensing and Hypoglycaemia

The best-established homeostatic process that involves brain glucose sensing is the defensive response to hypoglycaemia. In health, a fall in blood glucose triggers a series of physiological responses aimed at limiting the fall and restoring euglycaemia (see Ref. [1] for review). Counter-regulatory neurohumoral responses include the cessation of endogenous insulin release followed by release of glucagon, catecholamines (including adrenaline from the adrenal medulla and increased autonomic outflow with a rise in circulating noradrenaline from spillover from sympathetic synapses), growth hormone and triggering of the hypophyseal–adrenal axis [adrenocorticotrophin (ACTH) and glucocorticoids]. These changes tend to limit glucose utilization and attempt to increase hepatic glucose output, not only by hormonal effects, but also probably through direct autonomic innervation of the liver. Counter-regulatory neurohumoral changes are associated with the generation of ‘adrenergic’ warning symptoms such as sweating, shaking and heart palpitations. Importantly, hunger is also stimulated and consequent feeding will help restore and maintain euglycaemia.

If blood glucose falls sufficiently low, cognitive function starts to become impaired with a number of cognitive domains being affected – particularly those requiring attention, speed of response and judgement. Neuroglycopenic symptoms – for example, tingling of lips and irritability – are probable consequences of a fall in brain fuel. Because the brain is so dependent on circulating glucose to fuel metabolism and support function, it makes teleological sense for brain areas to be involved in monitoring blood glucose and triggering defences.

Evidence for a role for brain sensors in responses to hypoglycaemia came originally from studies using selective catheterization in dogs. Using this methodology, maintaining brain glucose during systemic hypoglycaemia resulted in a reduced neurohumoral reaction to the hypoglycaemic challenge, suggesting that it was the fall in glucose within the brain that triggered the processes leading to counter-regulation [2,3]. Elegant rat studies suggested an important role for the ventromedial nucleus of the hypothalamus (VMN), with local delivery of 2-deoxy glucose (2DG) to induce glucopenia, stimulating a counter-regulatory response. Also in keeping with a role for the VMN, lesioning of the VMN by sterotactic injection of ibotenic acid reduced counter-regulation. Similarly, maintenance of VMN glucose by locally delivered glucose during hypoglycaemia reduced counter-regulation [4–6]. Supporting a role for the brain stem, studies using 2DG or the alternative glucoprivic agent, 5-thioglucose, showed that hindbrain sensing of glucopenia was important [7–9]. Consistent with these data, a number of studies using more contemporary experimental manipulations have confirmed that a number of sites in both hypothalamus and brain stem are critical for triggering counter-regulatory responses. For example, mice lacking vesicular transporters for glutamate in VMN steroidogenic factor 1 (SF1) SF1 neurones have defective glucagon response to hypoglycaemia [10].

Importantly, in addition to brain sensing of hypoglycaemia, glucose sensors from the periphery, particularly portal vein sensors, may also play a significant role, especially for slowly developing hypoglycaemia [11–13], probably contributing information via vagal afferents into the dorsal vagal complex in the brain stem [14].

In diabetes, aggressive blood glucose lowering therapy increases risk of overshooting such that counter-regulatory responses become critical to preventing and/or limiting hypoglycaemia and restoring normal glucose. Described later, some patients develop deficits in counter-regulatory defences, putting them at greater risk of severe hypoglycaemia. A better understanding of the physiology of counter-regulation including how hypoglycaemia is sensed – whether glucokinase-mediated or not – and how these responses may become altered in diabetes could allow prevention of problematic hypoglycaemia.

## Brain Glucose Sensing and Pancreatic Insulin Secretion

Over the last decade, the role of the brain in controlling hepatic glucose flux, at least in rodent models, has been firmly established. In particular, there is a clear circuitry from the hypothalamus via vagal outflow to control glycaemia in response to locally detected changes in nutrients including glucose, and the circuitry has been well-described elsewhere [15].

A similar role for the brain in modulating glucose-stimulated insulin secretion has also been suggested. Although the pancreas and indeed isolated islets and *β*-cells are capable of detecting changes in blood glucose and altering insulin secretion appropriately, the pancreas, including islets, is richly innervated by sympathetic (splanchnic), parasympathetic (vagus) systems and non-autonomic innervation. It has long been known that the taste, smell and even sight of food can trigger insulin release, referred to as the cephalic phase of insulin secretion [16]. A number of studies have shown that altering neural metabolism experimentally can change islet hormone release, including insulin [17]. Stimulation of the vagus nerve increases pancreatic insulin output [18–20], whereas the net effect of splanchnic nerve stimulation is to reduce insulin and increase glucagon output [21]. A number of studies using retrograde tracers such as modified pseudorabies virus injected into the pancreas have mapped out the central brain connections to pancreas, including areas that contain fuel-sensing neurones such as the dorsal vagal complex in the brain stem and key hypothalamic nuclei (reviewed in Ref. [17]).

Supporting a role for direct brain glucose sensing in the facilitation of insulin release, elegant studies in catheterized rats have shown that a small glucose bolus (insufficient to result in a systemic rise in glucose) given directly into the carotid artery directed towards brain induced a peripheral insulin response. This was associated with activation of the hypothalamic paraventricular and arcuate nuclei. [22,23]. Recent data suggest that, in addition to effects on insulin secretion, brain glucose sensing acting via pancreatic innervation may control *β*-cell mass [24].

## Brain Glucose Sensing and Feeding/Energy Homeostasis

Although hypoglycaemia is a very potent stimulator of appetite, the role of brain glucose sensing at more physiological glucose levels remains unclear. Over 50 years ago, the glucostatic theory postulated that blood glucose levels were a physiological determinant of feeding. Suggested mechanisms were either in the initiation of eating, with small dips in blood glucose appearing to precede feeding and/or by the rise in blood glucose induced by feeding then acting as a satiety/meal termination signal.

Energy balance depends on intake but also on energy expenditure. Like other nutrients, glucose may also act centrally to generate thermogenesis [25], perhaps dependent on brain GLUT2-mediated glucose sensing (see below).

## Mechanisms of Brain Glucose Sensing

For all of the above processes to occur, brain nutrient sensors need to be able to detect rapidly and accurately changes in blood glucose, presumably by detecting corresponding changes in ambient glucose within the brain. Glucose-sensing neurones were first described in the 1960s, with two populations of neurones being either activated by glucose (glucose-excited – GE) or inhibited (originally referred to as glucose-sensitive but now globally labelled as glucose-inhibited – GI) [26,27]. GE and GI neurones have been identified in an increasing number of brain areas. Within the hypothalamus, both GE and GI neurons are found in the arcuate and paraventricular nuclei, with GI also particularly in the lateral hypothalamus and GE in the VMN [28]. GE and GI neurones are also found in the nucleus tracts solitarius and area postrema in the brain stem [29,30]. It appears probable that different subgroups of GE and GI neurones exist, with some operating at higher glucose values (‘high GE’ and ‘high GI’ neurones).

Although most work has focused on the metabolic-sensing potential of neurones, it is clear that there is a close metabolic coupling between surrounding glial cells and neurones [31]. This may be important in glucose sensing also, for example with glucose being taken up by astrocytes and metabolized to lactate, which is then transported across into neurones. In this situation, glucose-sensing neurones would be responding to lactate rather than glucose *per se*.

There are probably a number of mechanisms by which GE and GI neurones respond to glucose and/or other fuels such as lactate. In broad terms, most postulated sensing mechanisms are ‘metabolic’, that is they depend on glucose-entering cells and being metabolized, with fuel sensing then detecting the process or products of that metabolism. Alternatively, sensing may use non-metabolic signals. For example, glucose sensing using the electrogenic signal generated by active glucose transport by sodium-coupled glucose co-transporter (SGLT) 1/3 has been described [32]. It is also possible that glucose sensing may occur without glucose entry into cells – for example by tandem pore potassium channels or perhaps even by taste receptors [33]. In this review, we have described briefly some of the metabolic sensors postulated to be important.

A number of research groups have examined the analogies between pancreatic *β*-cell and brain glucose sensing. Peculiar features of the *β*-cell that allow it to detect glucose (at least in the canonical pathway) include ATP-gated potassium (K_ATP_) channels, the low-affinity glucose transporter GLUT2 and the low-affinity hexokinase, glucokinase.

The role of brain glucokinase is described later in this review. K_ATP_ channels are distributed widely in the brain, including in glucose-sensing areas. This wide distribution suggests that their role is broader than nutrient sensing – perhaps in protection of neurones against injurious insults. They have, however, been implicated in brain glucose sensing. Using a pharmacological approach, closing K_ATP_ channels by brain infusion of sulphonylureas attenuated responses to hypoglycaemia [34] and brain delivery of K_ATP_ channel openers increased hypoglycaemic counter-regulatory responses [35]. Mice deficient for the Kir6.2 subunit of K_ATP_ channels showed reduced responses in glucose-sensing neurones in the ventromedial hypothalamus and defective glucagon (but not adrenaline) responses to both hypoglycaemia and brain glucopenia. Of note, isolated islets from these mice studied *ex vivo* showed glucagon responses to glucose similar to those of wild-type mice [36].

Data suggest that hypothalamic levels of the inhibitory neurotransmitter γ-aminobutyric acid (GABA) may act downstream of K_ATP_ channels to modulate glucagon responses to hypoglycaemia. Closing or opening K_ATP_ channels in the VMN resulted in increased or decreased VMN GABA levels, respectively, associated with suppression or amplification of glucagon and other counter-regulatory responses to hypoglycaemia [37,38]. Microperfusion of glucose into the VMN of rats suppressed glucagon and adrenaline responses to hypoglycaemia and altered VMN GABA with these effects of VMH glucose being blocked by GABA A antagonism [39].

The low-affinity transporter, GLUT2, has also been identified within brain regions. Using an elegant transgenic mouse model combining GLUT2 knockout (with re-expression of the alternative transporter GLUT1 in pancreatic *β*-cells to maintain insulin secretion), Thorens' group found reduced glucagon responses to glucoprivation [40]. Glucagon responses to low glucose were restored by brain re-expression of GLUT2, but interestingly in glial rather than neuronal cells [41]. Parenthetically, the insulin-responsive transporter GLUT 4 and indeed insulin receptors have been identified in VMN GE and GI neurones although the significance for metabolic homeostasis remains to be fully determined [42].

A number of ‘non-*β*-cell’ sensing mechanisms dependent on intracellular metabolism of glucose have also been implicated in brain glucose sensing. Perhaps the best characterized is the cellular energy sensor AMP-activated protein kinase (AMPK), which is found widely in the brain, including in glucose-responsive cells [43]. Targeted genetic knockdown of AMPK in rat VMN suppressed glucagon responses to hypoglycaemia, whereas pharmacological activation of VMN AMPK amplified responses in rats with impaired counter-regulation [44,45].

## Distribution of Glucokinase in the Brain

Approaches taken to identify the presence and distribution of glucokinase in the brain have largely looked for messenger RNA (mRNA) (RT-PCR and *in situ* hybridization) and activity assays measuring low-affinity glucose phosphorylation activity. Identifying glucokinase protein reliably has been more challenging, perhaps because of low levels of expression.

Studies using transgenic mice with human growth hormone expressed under the glucokinase promotor confirmed glucokinase expression in the brain, but detailed characterization of glucokinase-containing neurones was limited by a relatively low resolution [46]. This was also true for *in situ* hybridization studies with or without dual immunohistochemistry and RT-PCR work that identified some dual staining with candidate neuropeptides. However, full characterization was difficult [47,48]. Intriguingly, glucokinase mRNA has also been identified in tanycytes, raising the possibility that this population of glial cells are involved in metabolic sensing [49].

Stanley et al. have recently reported a comprehensive characterization of glucokinase-expressing cells in the brain [50]. To do so, they created a mouse line expressing cre-recombinase under control of the neuronal glucokinase promotor and crossed mice with a line expressing an EGFP-tagged ribosomal construct. This allowed them to examine the distribution of glucokinase in the brain and also to perform transcriptional profiling of glucokinase-expressing cells. Using this model, glucokinase was identified in hypothalamic and limbic regions of the brain, with many of the areas having been identified (albeit with less resolution) by previous studies. They also examined and quantified responses to either 2DG or glucose and identified activation measured by c-fos expression in glucokinase cells.

Perhaps the most insightful part of their work came from transcriptional profiling. Hypothalamic enrichment was identified for a number of neuropeptides that might have been predicted to be found in glucose-sensing cells [orexin, pro-opiomelanocortin (POMC), neuropeptide Y (NPY) and agouti-related peptide (AGRP)]. Dual immunohistochemistry confirmed that glucokinase was found in a subset of the corresponding cell populations with 36% of lateral hypothalamic orexin neurones (GI), 20% of POMC (GE) and 28% of NPY/AGRP (GI) neurones. Importantly, this emphasizes that there is likely to be heterogeneity within these cellular populations with some either using different fuel-sensing apparatus or perhaps not possessing the ability to sense glucose directly. Also of note, approximately one third of glucokinase-expressing cells in the brain identified by this approach were glial rather than neuronal.

There was a marked enrichment in growth-hormone-releasing hormone (GHRH)-containing neurones. Subsequent dual immunohistochemistry and patch clamp studies confirmed that the majority of GHRH neurones contained glucokinase and that they were GI with activity increasing as blood glucose fell. Growth hormone is a part of the counter-regulatory hormonal response to hypoglycaemia, and prior to this work, the mechanisms by which this was triggered were unclear. It appears probable that this occurs via direct glucose sensing by GHRH neurones utilizing glucokinase.

A paradox remains for *β*-cell-type adaptations such as glucokinase and GLUT2, namely why brain would use low-affinity mechanisms. Brain glucose values undoubtedly vary from region to region, but are probably 10 to 30% of those observed in the circulation. If so, one would predict that glucokinase and GLUT2 would be largely inactive at ambient glucose levels, and certainly so during hypoglycaemia. The question remains unresolved. Although brain glucose sensors are clustered close to circumventricular organs such as the median eminence (hypothalamus) and area postrema (brain stem) where the blood–brain barrier is deficient, there is currently little evidence that this alters ambient glucose levels in the sensing areas *per se*.

## Altering Brain Glucokinase Activity Experimentally

*Ex vivo*, a number of studies using fresh brain slices and/or primary hypothalamic cultures have identified that glucokinase is part of the sensing mechanism for at least a subset of GE and GI hypothalamic neurones [42,51–53]. Knockdown of glucokinase by interfering RNA in VMN preparations largely abolished GE and GI glucose-sensing activities, whereas glucokinase activation amplified both GE and GI responses. Extending this work *in vivo*, infusion of GK activators into the VMN reduced glucagon response to hypoglycaemia in rats. Similarly, inhibiting glucokinase with alloxan or short hairpin RNA (shRNA) knockdown increased responses – predominantly adrenaline – to hypoglycaemic challenges [54]. Outside the hypothalamus, glucokinase-dependent glucose sensing was also identified in the medial amygdala nucleus with a population of glucokinase-containing sensing neurones utilizing urocortin-mediated connections to the basomedial hypothalamus exerting a control loop to increase or decrease counter-regulatory responses to hypoglycaemia [55]. Using third ventricle infusion of pharmacological inhibitors of glucokinase, we were able to stimulate feeding responses to glucoprivation [56]. This was associated with activation of NPY and orexigenic cells [57].

One study has examined counter-regulatory hormone responses in humans with glucokinase mutations leading to ‘MODY-2’ monogenic diabetes [58]. Glucagon responses to hypoglycaemia were increased, although it is unclear whether this was because of pancreatic or brain glucokinase in subjects with a whole-body glucokinase activity alteration.

Interesting data examining the effect of the proapoptotic protein, BCl-associated death promotor (BAD) may also reflect a role for brain glucokinase in responses to hypoglycaemia. In the *β*-cell, BAD alters glucose levels by altering insulin secretion by at least two different mechanisms depending on its phosphorylation status – an interaction with glucokinase and by its proapoptotic actions. Acute genetic knockdown of BAD in the brain impaired the glucagon response to systemic glucoprivation in mice, although whether this was mediated through altered brain GK activity remains unclear [59].

We have also examined whether brain glucokinase-mediated glucose sensing is important in mediating insulin responses to a rise in blood glucose [60]. We used intravenous glucose tolerance tests in rats with delivery of test substances into the base of the third ventricle. We first examined delivery of glucose, reasoning that if brain sensing of glucose was important for dealing with a systemic glucose load, that we would see an increased efficiency of dealing with glucose challenge. In keeping with this, we found that glucose handling was improved, particularly early-phase insulin over the first 10 min after intravenous glucose delivery. Consistent with a role for brain glucose sensing in facilitating insulin release, early-phase insulin release was stimulated.

We then examined whether brain glucokinase contributed to this observation, delivering glucokinase inhibitors into the third ventricle of rats prior to and during intravenous glucose bolus delivery. Again, in keeping with our hypothesis, intracerebroventricular glucosamine or mannoheptulose, an alternative inhibitor, reduced glucose tolerance associated with a decrease in insulin release over the first few minutes.

We have also examined the effects of pharmacological inhibition of brain glucokinase on feeding reponses. In keeping with a role for brain glucokinase in central glucose sensing, central delivery of glucosamine or mannoheptulose rapidly triggered robust feeding responses in rats [56].

## Changes in Glucose Sensing in Diabetes and Obesity?

A subset of patients with diabetes – particularly but not only those with type 1 diabetes – develop deficiencies in their counter-regulatory-defensive responses in the face of falling blood glucose. Neurohumoral responses are delayed and diminished, and the associated warning symptoms are also reduced. This altered physiology is associated clinically with a significantly increased risk of suffering from severe hypoglycaemia. Given the evidence for brain glucose sensing playing a key role in triggering counter-regulation (as discussed earlier), the unproven assumption is that this loss of counter-regulation or loss of warning symptoms is a consequence of altered glucose sensing and/or adaptations in the circuitry occurring very close to the site of glucose sensing.

The exact mechanisms remain to be fully determined and it is unclear whether glucokinase may play a role in this. For example, in juvenile rats, glucokinase expression within the VMN was increased after hypoglycaemia and associated with a shift in glucose responsiveness of GE neurons [61]. In contrast, we found no change in hypothalamic glucokinase expression following recurrent hypoglycaemia in rat brains, but rather an increase in hexokinase expression [62]. Regardless of whether involved in the pathoaetiology, it is possible that targeting brain glucokinase might alter counter-regulatory responses to hypoglycaemia for good or bad. For example, one might predict a theoretical risk of reducing counter-regulation with glucokinase activation, a concern given that a number of pharmaceutical agents are in development for treating diabetes through activation of peripheral (*β*-cell and/or liver) GK. Some reassurance comes from studies in humans in which a single dose of a glucokinase activator had no obvious central effect on counter-regulatory responses to hypoglycaemia, with suppressed glucagon responses probably related to a local paracrine effect from altered insulin levels [63].

There is no compelling evidence for an effect of brain glucokinase on appetite and energy balance. As described above, we found that experimental inhibition of glucokinase in the rat brain stimulated glucoprivic feeding [56], but it is not clear how much overlap there is (if any) between feeding in response to a lack of glucose (a potential emergency situation) and general appetitive behaviour. We await more specific tools to manipulate brain glucokinase – perhaps targeted conditional transgenic murine models – to examine in a more definitive way whether brain glucokinase plays a broader role in energy metabolism.

## Summary: Brain Glucokinase and Glucose Sensing

In summary, it appears probable that brain glucokinase is present in a variety of glucose-sensing cells – possibly both glial and neuronal. Intriguingly, it appears that this includes both GE cells – analogous to the pancreatic *β*-cell – but also GI neurones suggesting other downstream-mediating effects. It is tempting to speculate that GI neurones may be broadly analogous to pancreatic α-cells, but perhaps safest to assume that this all illustrates that brain glucose sensing shares some, but not all, features of those mechanisms identified elsewhere in the body.

## References

[1] Evans ML, Amiel SA, Wass J, Shalet S (2002). Hypoglycaemia. Oxford Textbook of Endocrinology.

[2] Frizzell RT, Jones EM, Davis SN (1993). Counterregulation during hypoglycemia is directed by widespread brain regions. Diabetes.

[3] Biggers DW, Myers SR, Neal D (1989). Role of brain in counterregulation of insulin-induced hypoglycemia in dogs. Diabetes.

[4] Borg WP, Sherwin RS, During MJ, Borg MA, Shulman GI (1995). Local ventromedial hypothalamus glucopenia triggers counterregulatory hormone release. Diabetes.

[5] Borg WP, During MJ, Sherwin RS, Borg MA, Brines ML, Shulman GI (1994). Ventromedial hypothalamic lesions in rats suppress counterregulatory responses to hypoglycemia. J Clin Invest.

[6] Borg MA, Sherwin RS, Borg WP, Tamborlane WV, Shulman GI (1997). Local ventromedial hypothalamus glucose perfusion blocks counterregulation during systemic hypoglycemia in awake rats. J Clin Invest.

[7] Ritter RC, Slusser PG, Stone S (1981). Glucoreceptors controlling feeding and blood glucose: location in the hindbrain. Science.

[8] Ritter S, Bugarith K, Dinh TT (2001). Immunotoxic destruction of distinct catecholamine subgroups produces selective impairment of glucoregulatory responses and neuronal activation. J Comp Neurol.

[9] Ritter S, Dinh TT, Zhang Y (2000). Localization of hindbrain glucoreceptive sites controlling food intake and blood glucose. Brain Res.

[10] Tong Q, Ye C, McCrimmon RJ (2007). Synaptic glutamate release by ventromedial hypothalamic neurons is part of the neurocircuitry that prevents hypoglycemia. Cell Metab.

[11] Hevener AL, Bergman RN, Donovan CM (2000). Portal vein afferents are critical for the sympathoadrenal response to hypoglycemia. Diabetes.

[12] Saberi M, Bohland M, Donovan CM (2008). The locus for hypoglycemic detection shifts with the rate of fall in glycemia: the role of portal-superior mesenteric vein glucose sensing. Diabetes.

[13] Matveyenko AV, Bohland M, Saberi M, Donovan CM (2007). Portal vein hypoglycemia is essential for full induction of hypoglycemia-associated autonomic failure with slow-onset hypoglycemia. Am J Physiol Endocrinol Metab.

[14] Delaere F, Akaoka H, De Vadder F, Duchampt A, Mithieux G (2013). Portal glucose influences the sensory, cortical and reward systems in rats. Eur J Neurosci.

[15] Pocai A, Lam TK, Gutierrez-Juarez R (2005). Hypothalamic K(ATP) channels control hepatic glucose production. Nature.

[16] Teff KL, Mattes RD, Engelman J, Mattern J (1993). Cephalic-phase insulin in obese and normal-weight men: relation to postprandial insulin. Metabolism.

[17] Osundiji MA, Evans ML (2013). Brain control of insulin and glucagon secretion. Endocrinol Metab Clin North Am.

[18] Kaneto A, Kosaka K, Nakao K (1967). Effects of stimulation of the vagus nerve on insulin secretion. Endocrinology.

[19] Findlay JA, Gill JR, Lever JD, Randle PJ, Spriggs TL (1969). Increased insulin output following stimulation of the vagal supply to the perfused rabbit pancreas. J Anat.

[20] Frohman LA, Ezdinli EZ, Javid R (1967). Effect of vagotomy and vagal stimulation on insulin secretion. Diabetes.

[21] Bloom SR, Edwards AV (1975). Pancreatic endocrine responses to stimulation via the sympathetic innervation. J Physiol.

[22] Guillod-Maximin E, Lorsignol A, Alquier T, Pénicaud L (2004). Acute intracarotid glucose injection towards the brain induces specific c-fos activation in hypothalamic nuclei: involvement of astrocytes in cerebral glucose-sensing in rats. J Neuroendocrinol.

[23] Leloup C, Magnan C, Benani A (2006). Mitochondrial reactive oxygen species are required for hypothalamic glucose sensing. Diabetes.

[24] Tarussio D, Metref S, Seyer P (2014). Nervous glucose sensing regulates postnatal *β* cell proliferation and glucose homeostasis. J Clin Invest.

[25] Le Feuvre RA, Woods AJ, Stock MJ, Rothwell NJ (1991). Effects of central injection of glucose on thermogenesis in normal, VMH-lesioned and genetically obese rats. Brain Res.

[26] Anand BK, Chhina GS, Sharma KN, Dua S, Singh B (1964). Activity of single neurons in the hypothalamic feeding centers: effect of glucose. Am J Physiol.

[27] Oomura Y, Yoshimatsu H (1984). Neural network of glucose monitoring system. J Auton Nerv Syst.

[28] Silver IA, Erecinska M (1998). Glucose-induced intracellular ion changes in sugar-sensitive hypothalamic neurons. J Neurophysiol.

[29] Adachi A, Shimizu N, Oomura Y, Kobashi M (1984). Convergence of hepatoportal glucose-sensitive afferents signal to glucose sensitive units within the nucleus of the solitary tract. Neurosci Lett.

[30] Dallaporta M, Himmi T, Perrin J, Orsini JC (1999). A solitary tract nucleus sensitivity to moderate changes in glucose level. Neuroreport.

[31] Magistretti PJ, Pellerin L, Rothman DL, Shulman RG (1999). Energy on demand. Science.

[32] O'Malley D, Reimann F, Simpson AK, Gribble FM (2006). Sodium-coupled glucose cotransporters contribute to hypothalamic glucose sensing. Diabetes.

[33] Burdakov D, Jensen LT, Alexopoulos H (2006). Tandem-pore K+ channels mediate inhibition of orexin neurons by glucose. Neuron.

[34] Evans ML, McCrimmon RJ, Flanagan DE (2004). Hypothalamic ATP-sensitive K+ channels play a key role in sensing hypoglycemia and triggering counterregulatory epinephrine and glucagon responses. Diabetes.

[35] McCrimmon RJ, Evans ML, Fan X (2005). Activation of ATP-sensitive K+ channels in the ventromedial hypothalamus amplifies counterregulatory hormone responses to hypoglycemia in normal and recurrently hypoglycemic rats. Diabetes.

[36] Miki T, Liss B, Minami K (2001). ATP-sensitive K+ channels in the hypothalamus are essential for the maintenance of glucose homeostasis. Nat Neurosci.

[37] Chan O, Lawson M, Zhu W, Beverly JL, Sherwin RS (2007). ATP-sensitive K(+) channels regulate the release of GABA in the ventromedial hypothalamus during hypoglycemia. Diabetes.

[38] Chan O, Zhu W, Ding Y, McCrimmon RJ, Sherwin RS (2006). Blockade of GABA(A) receptors in the ventromedial hypothalamus further stimulates glucagon and sympathoadrenal but not the hypothalamo-pituitary-adrenal response to hypoglycemia. Diabetes.

[39] Zhu W, Czyzyk D, Paranjape SA (2010). Glucose prevents the fall in ventromedial hypothalamic GABA that is required for full activation of glucose counterregulatory responses during hypoglycemia. Am J Physiol Endocrinol Metab.

[40] Thorens B, Guillam MT, Beermann F, Burcelin R, Jaquet M (2000). Transgenic reexpression of GLUT1 or GLUT2 in pancreatic beta cells rescues GLUT2-null mice from early death and restores normal glucose-stimulated insulin secretion. J Biol Chem.

[41] Marty N, Dallaporta M, Foretz M (2005). Regulation of glucagon secretion by glucose transporter type 2 (glut2) and astrocyte-dependent glucose sensors. J Clin Invest.

[42] Kang L, Routh VH, Kuzhikandathil EV, Gaspers LD, Levin BE (2004). Physiological and molecular characteristics of rat hypothalamic ventromedial nucleus glucosensing neurons. Diabetes.

[43] Beall C, Hamilton DL, Gallagher J (2012). Mouse hypothalamic GT1-7 cells demonstrate AMPK-dependent intrinsic glucose-sensing behaviour. Diabetologia.

[44] McCrimmon RJ, Shaw M, Fan X (2008). Key role for AMP-activated protein kinase in the ventromedial hypothalamus in regulating counterregulatory hormone responses to acute hypoglycemia. Diabetes.

[45] McCrimmon RJ, Fan X, Cheng H (2006). Activation of AMP-activated protein kinase within the ventromedial hypothalamus amplifies counterregulatory hormone responses in rats with defective counterregulation. Diabetes.

[46] Jetton TL, Liang Y, Pettepher CC (1994). Analysis of upstream glucokinase promoter activity in transgenic mice and identification of glucokinase in rare neuroendocrine cells in the brain and gut. J Biol Chem.

[47] Lynch RM, Tompkins LS, Brooks HL, Dunn-Meynell AA, Levin BE (2000). Localization of glucokinase gene expression in the rat brain. Diabetes.

[48] Dunn-Meynell AA, Routh VH, Kang L, Gaspers L, Levin BE (2002). Glucokinase Is the likely mediator of glucosensing in both glucose-excited and glucose-inhibited central neurons. Diabetes.

[49] Millán C, Martínez F, Cortés-Campos C (2010). Glial glucokinase expression in adult and post-natal development of the hypothalamic region. ASN Neuro.

[50] Stanley S, Domingos A, Kelly L (2013). Profiling of glucose-sensing neurons reveals that GHRH neurons are activated by hypoglycemia. Cell Metab.

[51] Kang L, Dunn-Meynell AA, Routh VH (2006). Glucokinase is a critical regulator of ventromedial hypothalamic neuronal glucosensing. Diabetes.

[52] Yang XJ, Kow LM, Funabashi T, Mobbs CV (1999). Hypothalamic glucose sensor: similarities to and differences from pancreatic beta-cell mechanisms. Diabetes.

[53] Yang XJ, Kow LM, Pfaff DW, Mobbs CV (2004). Metabolic pathways that mediate inhibition of hypothalamic neurons by glucose. Diabetes.

[54] Levin BE, Becker TC, Eiki J-I, Zhang BB, Dunn-Meynell AA (2008). Ventromedial hypothalamic glucokinase is an important mediator of the counterregulatory response to insulin-induced hypoglycemia. Diabetes.

[55] Zhou L, Podolsky N, Sang Z (2010). The medial amygdalar nucleus: a novel glucose-sensing region that modulates the counterregulatory response to hypoglycemia. Diabetes.

[56] Osundiji MA, Zhou L, Shaw J (2010). Brain glucosamine boosts protective glucoprivic feeding. Endocrinology.

[57] Zhou L, Yueh CY, Lam DD (2011). Glucokinase inhibitor glucosamine stimulates feeding and activates hypothalamic neuropeptide Y and orexin neurons. Behav Brain Res.

[58] Guenat E, Seematter G, Philippe J, Temler E, Jequier E, Tappy L (2000). Counterregulatory responses to hypoglycemia in patients with glucokinase gene mutations. Diabetes Metab.

[59] Osundiji MA, Godes ML, Evans ML, Danial NN BAD modulates counterregulatory responses to hypoglycemia and protective glucoprivic feeding. PLoS One.

[60] Osundiji MA, Lam DD, Shaw JS (2011). Brain glucose sensors play a significant role in the regulation of pancreatic glucose-stimulated insulin secretion. Diabetes.

[61] Kang L, Sanders NM, Dunn-Meynell AA (2008). Prior hypoglycemia enhances glucose responsiveness in some ventromedial hypothalamic glucosensing neurons. Am J Physiol Regul Integr Comp Physiol.

[62] Osundiji MA, Hurst P, Moore SP (2011). Recurrent hypoglycemia increases hypothalamic glucose phosphorylation activity in rats. Metabolism.

[63] Norjavaara E, Ericsson H, Sjöberg F (2012). Glucokinase activators AZD6370 and AZD1656 do not affect the central counterregulatory response to hypoglycemia in healthy males. J Clin Endocrinol Metab.

